# Extracellular Heat Shock Protein (Hsp)70 and Hsp90α Assist in Matrix Metalloproteinase-2 Activation and Breast Cancer Cell Migration and Invasion

**DOI:** 10.1371/journal.pone.0018848

**Published:** 2011-04-14

**Authors:** Jessica D. Sims, Jessica McCready, Daniel G. Jay

**Affiliations:** 1 Department of Cellular and Molecular Physiology, Sackler School of Biomedical Sciences, Tufts University School of Medicine, Boston, Massachusetts, United States of America; 2 Department of Anatomy and Cellular Biology, Sackler School of Biomedical Sciences, Tufts University School of Medicine, Boston, Massachusetts, United States of America; University of Bergen, Norway

## Abstract

Breast cancer is second only to lung cancer in cancer-related deaths in women, and the majority of these deaths are caused by metastases. Obtaining a better understanding of migration and invasion, two early steps in metastasis, is critical for the development of treatments that inhibit breast cancer metastasis. In a functional proteomic screen for proteins required for invasion, extracellular heat shock protein 90 alpha (Hsp90α) was identified and shown to activate matrix metalloproteinase 2 (MMP-2). The mechanism of MMP-2 activation by Hsp90α is unknown. Intracellular Hsp90α commonly functions with a complex of co-chaperones, leading to our hypothesis that Hsp90α functions similarly outside of the cell. In this study, we show that a complex of co-chaperones outside of breast cancer cells assists Hsp90α mediated activation of MMP-2. We demonstrate that the co-chaperones Hsp70, Hop, Hsp40, and p23 are present outside of breast cancer cells and co-immunoprecipitate with Hsp90α *in vitro* and in breast cancer conditioned media. These co-chaperones also increase the association of Hsp90α and MMP-2 *in vitro*. This co-chaperone complex enhances Hsp90α-mediated activation of MMP-2 *in vitro*, while inhibition of Hsp70 in conditioned media reduces this activation and decreases cancer cell migration and invasion. Together, these findings support a model in which MMP-2 activation by an extracellular co-chaperone complex mediated by Hsp90α increases breast cancer cell migration and invasion. Our studies provide insight into a novel pathway for MMP-2 activation and suggest Hsp70 as an additional extracellular target for anti-metastatic drug development.

## Introduction

Breast cancer is the most commonly diagnosed cancer among women and is second only to lung cancer in cancer-related deaths [1]. There are a variety of treatments for primary breast tumors ranging from chemotherapies to tumor resection, but once breast cancer has metastasized, there is a poor rate of patient survival [2]. There are currently no therapies that limit metastasis. Obtaining a better understanding of the factors and mechanisms that regulate breast cancer cell migration and invasion is crucial to the development of treatments that limit breast cancer metastasis. Extracellular proteins are thought to play an important role in regulating cancer cell migration and invasion, [3], [4] and due to their accessibility, provide good targets for drug development. To explore this role, our lab conducted a functional proteomic screen for extracellular proteins that are essential for invasion in fibrosarcoma cells. One protein that was identified in the screen was extracellular heat shock protein 90α (Hsp90α).

Hsp90α is a highly conserved and abundant protein, constituting about 1% of the total intracellular protein [5], [6]. In the cytoplasm, Hsp90α has over 200 interacting proteins (hereafter referred to as “client proteins”) [7] and it commonly functions in concert with various co-chaperones including Hsp70, Hsp40, Hop, Hip, and p23. These proteins form a complex that binds to client proteins and assists in their folding or activation. Briefly, Hsp70 and Hsp40 form a complex with the client protein. This complex binds to Hsp90α using Hop as a scaffold. ATP binds to Hsp90α, initiating a conformation change that enables p23 to bind to Hsp90α and causes Hsp70, Hsp40, and Hop to disassociate from the complex. This facilitates the folding and activation of the client protein, which is then released from the complex [5], [8].

Many studies have focused on the intracellular role of Hsp90α in tumorigenesis, [9], [10], [11] but recently its extracellular role in migration and invasion is beginning to be elucidated. Hsp90α is exported from cancer cells via exosomes and contributes to breast cancer cell migration [12]. We, and others, have demonstrated that extracellular Hsp90α enhances breast cancer cell invasiveness through MMP-2 activation [13] as well as other client proteins including ErbB2 and plasmin [7], [12]. The mechanism of how Hsp90α acts in the activation of these client proteins is not known. We focus here on MMP-2 due to its well established role in invasion and migration [14], [15].

MMP-2 is a zinc-dependent endopeptidase and a member of the metalloproteinase family, which degrades various components of the extracellular matrix [16], [17]. MMP-2 has been shown to be important in development and cell motility, and has a well-documented role in cancer metastasis [14], [15]. MMP-2 is secreted from the cell as an inactive zymogen and, once outside, it is activated through cleavage of its pro-domain [14]. MMP-2 is thought to be primarily activated by the membrane type 1 matrix metalloproteinase (MT1-MMP) and tissue inhibitors of metalloproteinases (TIMPs), but has also been shown to be activated in the absence of MT1-MMP, suggesting the presence of alternative modes of activation such as auto-activation [18].

Based on its intracellular function, we hypothesized that extracellular Hsp90α enhances breast cancer migration and invasion by activating MMP-2 with the assistance of the co-chaperones, Hsp70, Hop, Hsp40, and p23. In this paper, we demonstrate that these co-chaperones are secreted from breast cancer cells and that they physically interact with Hsp90α, both as recombinant proteins and in breast cancer cell conditioned media. Additionally, the presence of these co-chaperones increases the interaction of Hsp90α and MMP-2 *in vitro*. We show that Hsp90α, Hsp70, Hsp40, Hop, and p23 by themselves do not cleave and activate pro-MMP-2, but enhance auto-activation. This set of co-chaperones is similar to the minimal complex needed for the activation of intracellular proteins such as the steroid hormone receptor [19]. We also provide evidence that Hsp70, as part of the above complex, is important in the activation of MMP-2 in cancer cell conditioned media. The inhibition of extracellular Hsp70 reduces MMP-2 activation and decreases cancer cell migration and invasion, implicating this co-chaperone as an additional extracellular therapeutic target.

## Materials and Methods

### Antibodies and Reagents

All antibodies were purchased from Assay Designs (MI), except for anti-MMP-2, which was purchased from R&D (MN), and anti-fibronectin which was purchased from BD (NJ). All recombinant proteins were purchased from Assay Designs except for pro-MMP-2, which was purchased from EMD (NJ). Batimastat was purchased from Tocris Biosciences (MO) and 17-AAG was purchased from R&D (MN). Unless noted, all other reagents were purchased from Sigma (MO).

### Cell Culture

MDA-MB-231 and HT-1080 cells were obtained from ATCC and MDA-MB-231-s4175 cells were a kind gift from Dr. Joan Massagué. Cells were maintained in DMEM supplemented with 10% FBS, 1% non-essential amino acids, and 1% penicillin-streptomycin. SUM1315 cells were a kind gift from Dr. Charlotte Kuperwasser and were maintained in Hams F12 media supplemented with 5% FBS, 5 µg/mL insulin, 10 ng/ml epidermal growth factor (EGF) and 1% penicillin-streptomycin. All cells were grown at 37°C under 7.5% CO_2_.

### Preparation of Cell Lysates and Conditioned Media

To prepare lysates, cells were lysed in 750 µl lysis buffer (20 mM Tris pH 7.5, 1% Triton X-100, 3 µl/ml protease inhibitor cocktail (Sigma)), incubated for 30 minutes on ice and spun down at 4000 RPM (3300 g) on a table top centrifuge for 4 minutes. The supernatant was then collected and a Bicinchoninic Acid (BCA) protein assay was performed (Pierce, IL). To prepare conditioned media, 3 million cells were plated in a T-150 flask. Twenty-four hours after plating, cells were washed twice with HBSS (Gibco) and re-fed with 15 mL serum free DMEM with 1% non-essential amino acids. The conditioned media were collected 48 hours later and concentrated by centrifugation in centrifugal filters (Millipore, MA). Protein quantification was determined by BCA assay.

### Co-Immunoprecipitation and Western Blot Analysis

Recombinant proteins or conditioned media were pre-cleared with washed Protein G beads (Sigma) for 1 hour, spun down to pellet the beads, and the supernatant was collected. Antibody was added and incubated for 3 hours at 4°C with constant rotation. Protein G beads that had been pre-cleared for 3 hours with 1 mg/ml BSA and then washed 5 times with PBS, were added to the protein-antibody mixture and incubated for 1 hour at 4°C on a rotator. The beads were washed 5 times with lysis buffer, and the protein was eluted by boiling for 5 minutes in 4X sample buffer. The samples were run via SDS-PAGE on a blank gel, transferred to a nitrocellulose membrane, and blocked with antibodies against Hsp90α.

### Zymography/MMP-2 Activation

Pro-MMP-2 was activated by incubating recombinant MMP-2 (0.4 µg) in 4-Aminophenylmercuric Acetate (APMA) (1 mM in 0.1 M Tris-HCl) for 2 hours at 37°C. For *in vitro* Hsp90α activation of MMP-2, the indicated recombinant proteins and ATP or ATPγS were incubated in activation buffer (25 mM Tris pH 7.5, 50 mM KCl, 5 mM MgCl_2_, and 20 mM NaMoO_4_ [20]) for 30 minutes at 30°C. The samples were boiled for 5 minutes in 4X non-reducing sample buffer and run on a 10% polyacrylamide gel containing gelatin (4 mg/ml) [21], [22]. The gel was renatured in 2.5% Triton X-100 for 40 minutes at room temperature, washed in digestion buffer (0.1 M Tris pH 7.5, 50 mM CaCl_2_ and 1% NaN_3_) for 30 minutes and then incubated in fresh digestion buffer at 37°C for 18 hours. For activation of MMP-2 in conditioned media, conditioned media and untreated media were collected and concentrated. Aliquots of PBS, untreated media, and conditioned media (30 µl) were treated with the indicated inhibitors and then pro-MMP-2 (0.75 µg) was added. Samples were incubated with gentle agitation at room temperature for 5 minutes, boiled for 5 minutes in 4X reducing sample buffer and run on an SDS-PAGE gel, transferred to a nitrocellulose membrane, and blocked with antibodies against MMP-2. Densitometry was performed to determine the relative amount of active MMP-2 in both the zymograms and SDS-PAGE gels.

### FITC-Casein Assay

A FITC-Casein Assay (Sigma, MO) was performed according to the manufacturer's directions except for the substitution of the incubation buffer for buffer (A): 50 mM Tris–HCl, pH 7.5, 0.1 M NaCl, 10 mM CaCl_2_, and 0.1% Brij-35 [23]. In brief, pro-MMP-2 was activated with APMA for 2 hours as described above. Pro-MMP-2 was combined with the indicated recombinant proteins in activation buffer for 30 minutes at 30°C. The samples were then added to the indicated amount of buffer (A) and FITC-Casein substrate and allowed to incubate at 37°C in the dark for 1 hour. The reaction was then stopped with 150 ul of 0.6 N Trichloroacetic acid (TCA). The samples were centrifuged on a table top centrifuge for 10 minutes at 10,000 g. 2 ul of the supernatant was added to 200 ul of assay buffer in each well of black 96-well plate and read on a plate reader (excitation wavelength of 485 nm and an emission wavelength of 535 nm).

### Invasion Assay

2.5 million MDA-MB-231 or SUM1315 cells were plated on five T-75 flasks and incubated for 48 hours. Then 2×10^4^ MDA-MB-231 or SUM1315 cells were labeled with cell tracker orange (CMTMR, Invitrogen), treated with either 10 µg/ml 17AAG, 30 µM Vehicle (water), or 30 µM Methylene Blue, and added to each well of a porous membrane (pore size 8 µm (Neuroprobe, Gaithersburg, MD)) with a basement membrane barrier (0.3 µg/µl Matrigel (BD Biosciences, Bedford, MA)). The cells were incubated for 24 hours at 37°C under 7.5% CO_2_. The number of cells that had invaded into the matrigel and migrated through the pores of the membrane was quantified for each well with a fluorescence plate reader (excitation 544 nm, emission 590 nm).

### Depletion of Hsp90α and Hsp70 from conditioned media

Conditioned media was collected as described above and pre-cleared with Protein G beads for 1 hour. Antibodies against Hsp90α or Hsp70 were added to the pre-cleared conditioned media and incubated for 3 hours. Afterwards, Protein G beads were added to the conditioned media-antibody mixture and rotated at 4°C overnight. The beads were then spun down and the depleted supernatant was collected.

### Wound Healing

MDA-MB-231-s4175 cells were plated (1.5×10^5^/per chamber) in an 8-well chamber slide. The cells were incubated for 18 hours to allow formation of a monolayer. A wound was created in the monolayer with a 200 µl pipette tip. Cells were washed once with DMEM to remove any detached cells and pictures were taken of the wounds (0 hour time point). Serum-free DMEM, untreated conditioned medium obtained from MDA-231-s4175 cells, Hsp90α-depleted, Hsp70-depleted, or fibronectin-depleted (serves as a depletion control) conditioned media, or inhibitor-treated conditioned media was added to the wounded cells. The cells were incubated at 37°C under 7.5% CO_2_. Images were taken to determine the amount of cell movement from the wound edge at 16 hours (16 hour time point). Wound closure was quantified by measuring the area of the wound before and after the 16-hour incubation period.

### Cell Viability Assay

Plated 20,000 MDA-MB-231 cells per well in a 96-well plate for 24 hours before addition of increasing volumes of 17-AAG, SPS-771, or Rabbit IgG that was incubated for 24 hours. Cells were subjected to a Celltiter 96 Aqueous Non-radioactive Cell Proliferation Assay (Promega, WI). The number of viable cells was calculated as previously described [24].

## Results

### Hsp90α, MMP-2, and the co-chaperones Hsp70, Hop, Hsp40, and p23 are present in breast cancer cell conditioned media

**Figure 1:**
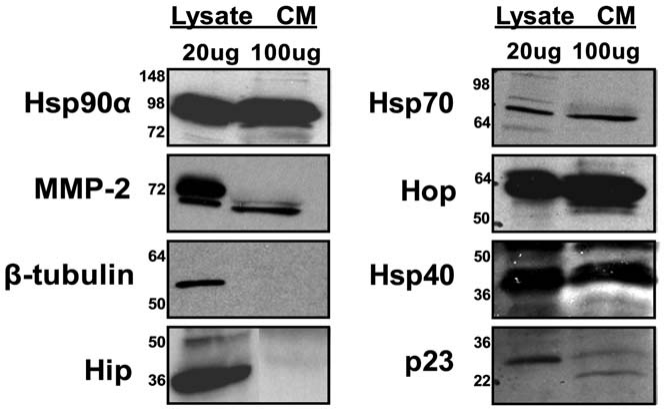
Hsp90α, MMP-2 and several co-chaperones are present in the conditioned media of breast cancer cells. Lysate and conditioned media were collected from MDA-MB-231 cells. 20 µg of protein was loaded into each lysate lane and 80 µg of protein was loaded into each conditioned media lane and immunoblotted for the Hsp90α, MMP-2, Hsp70, Hop, Hsp40, p23, Hip, and β-tubulin. The first six proteins were found to be present in both the cell lysate and in the conditioned media, while Hip was only found in the lysate. β-tubulin was used as a negative control. The molecular weight markers are indicated on the left.

Intracellular Hsp90α forms an activation complex with various co-chaperones including Hsp70, Hop, Hsp40, p23, and Hip [25]. This complex carries out the conformational changes necessary to fold or activate the client protein. We proposed that extracellular Hsp90α is functioning in a similar manner to intracellular Hsp90α; therefore we tested for the presence of the above co-chaperones in breast cancer cell conditioned media. We probed the extracellular media of the invasive breast cancer cell line, MDA-MB-231, for these co-chaperones. We observed Hsp70, Hop, Hsp40, and p23, but not Hip, in the conditioned media (Fig. 1). In addition to not finding Hip, the intracellular protein β-tubulin was not detected (Fig. 1), which indicates that the co-chaperones in the media were not the result of intracellular leakage from lysed or dying cells. All the co-chaperones were less abundant in conditioned media than in cell lysate. Hsp90α, Hsp70, and Hop were three times more abundant in the lysate than in the conditioned media, while MMP-2, Hsp40, and p23 were, respectively, 8, 6, and 10 times more abundant in the lysate than the conditioned media as determined by densitometry. These results, which demonstrate the presence of co-chaperones outside of invasive cancer cells, confirm and extend our previously published data that co-chaperones are present in conditioned media of fibrosarcoma cells [26], suggesting a general role for extracellular co-chaperones in cancer progression.

### Hsp90α interacts with Hsp70, Hop, Hsp40, p23, and MMP-2 both *in vitro* and in conditioned media

**Figure 2:**
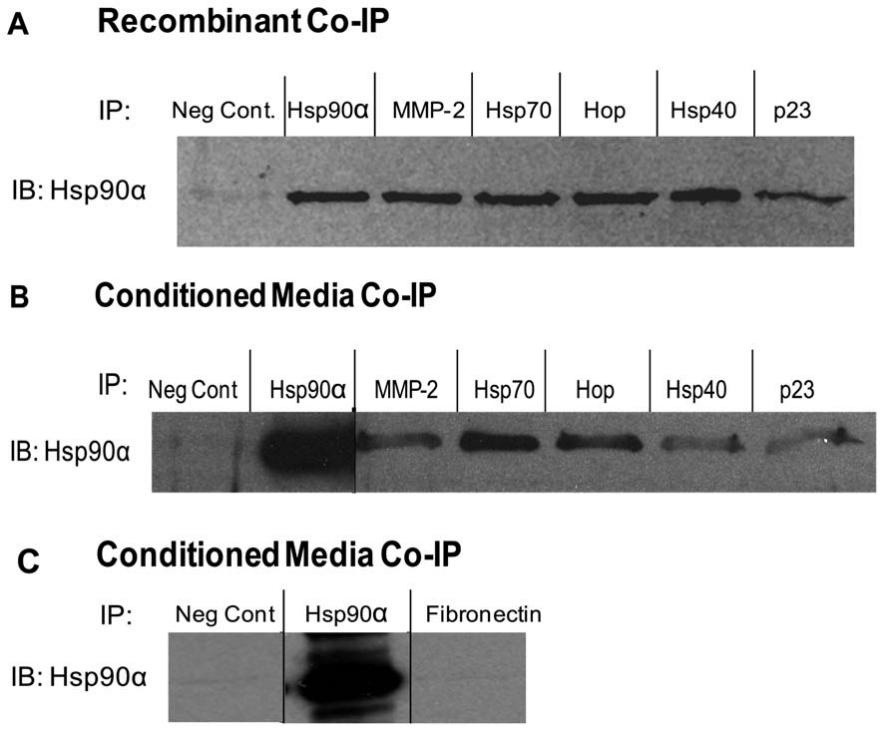
Hsp90α, MMP-2, Hsp70, Hop, Hsp40, and p23 interact *in vitro* and outside of the cell. (a) 1 µg Hsp90α, 0.5 µg MMP-2, 1 µg Hsp70, 0.25 µg Hop, 0.1 µg Hsp40, and 0.25 µg p23 recombinant proteins were combined and the indicated IP antibody was added to the proteins. The protein complexes were pulled down using Protein G beads. The immunoprecipitate was then run on an SDS-PAGE and blotted for Hsp90α. (b and c) Conditioned media was collected from MDA-MB-231 cells and the indicated IP antibody was added to equal volumes of conditioned media. The resulting complex was then pulled down with Protein G beads and run on an SDS-PAGE and blotted for Hsp90α.

To determine whether these extracellular co-chaperones can interact with Hsp90α, *in vitro* interaction was tested by co-immunoprecipitation with recombinant proteins and antibodies against Hsp70, Hop, Hsp40, p23, and MMP-2. Hsp90α was found to co-immunoprecipitate with each of these recombinant proteins (Fig. 2a). To determine if extracellular co-chaperones interact with Hsp90α *in culture*, interaction was tested in conditioned media from MDA-MB-231 breast cancer cells. Confirming our previous findings [13] and our *in vitro* results, Hsp90α interacted with MMP-2 in MDA-MB-231 conditioned media. Hsp90α also interacted with Hsp70, Hop, Hsp40, and p23 (Fig. 2b). To further ensure that the observed interaction was specific, co-immunoprecipitation with Hsp90α was tested for fibronectin, a protein detected outside of MDA-MB-231 cells that is not known to interact with Hsp90α. As can be seen in Fig. 2c, fibronectin did not co-immunoprecipitate with Hsp90α. Together, these findings show that Hsp90α forms a complex with Hsp70, Hop, Hsp40, and p23, in addition to MMP-2, both *in vitro* and in breast cancer cell media [5], [8].

### Hsp70, Hop, Hsp40, and p23 increase the interaction between Hsp90α and MMP-2, enhancing MMP-2 activation

**Figure 3:**
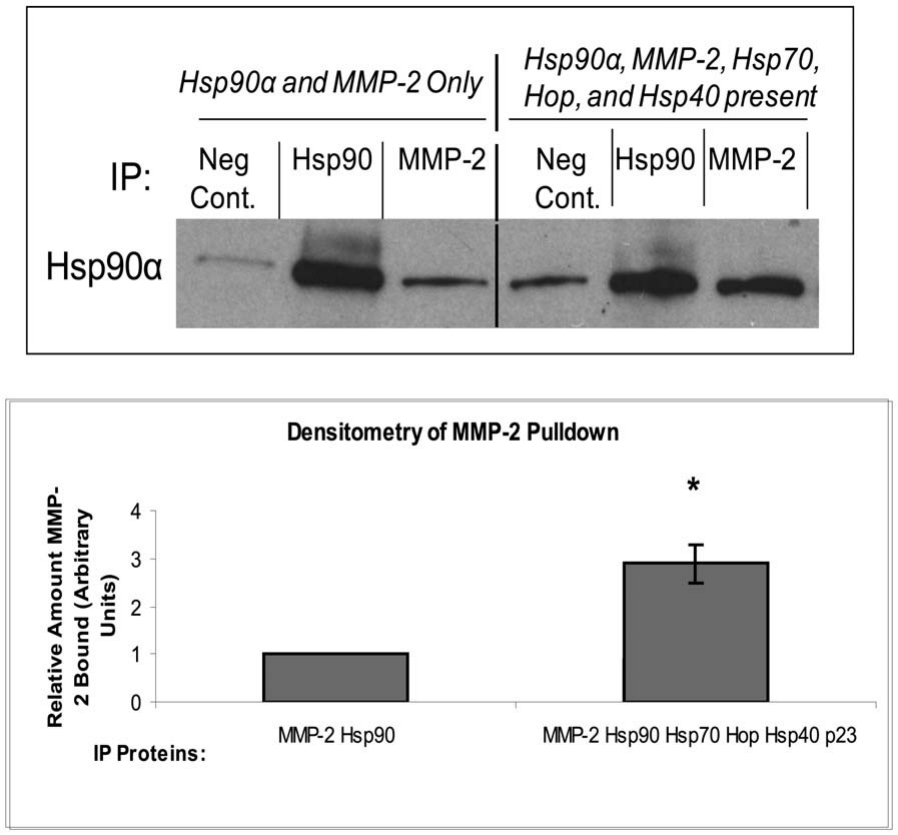
The presence of Hsp70, Hop, Hsp40, and p23 increase the interaction between Hsp90α and MMP-2. Using recombinant proteins, two co-immunoprecipitations were performed, one with Hsp90α and MMP-2 alone and one with Hsp90α, MMP-2, Hsp70, Hop, Hsp40, and p23 present using the same quantities as in Fig. 2. Hsp90α and MMP-2 primary antibody were used to pull down Hsp90α and then the resulting proteins were run on an SDS-PAGE and blotted for Hsp90α. The bands were then subject to densitometry to determine the relative interaction between Hsp90α and MMP-2 with and without the presence of the co-chaperones. Experiments were repeated three times. P-value<0.001.

Intracellular Hsp90α relies on co-chaperones to recruit its client proteins and to assist in the formation of an activation complex [27]. Consequently, the interaction between Hsp90α and its client proteins increases in the presence of these co-chaperones [5]. To test if the co-chaperones Hsp70, Hop, Hsp40, and p23 enhance the interaction between Hsp90α and MMP-2, we performed co-immunoprecipitations with Hsp90α and MMP-2 either alone or in the presence of the four co-chaperones that we previously determined were outside of the cell. Figure 3 shows that addition of the co-chaperones increased the interaction between Hsp90α and MMP-2 by 2.9 fold (Fig. 3, p<0.01). We observed a small amount of non-specific binding, which was accounted for in the densitometry calculations.

**Figure 4:**
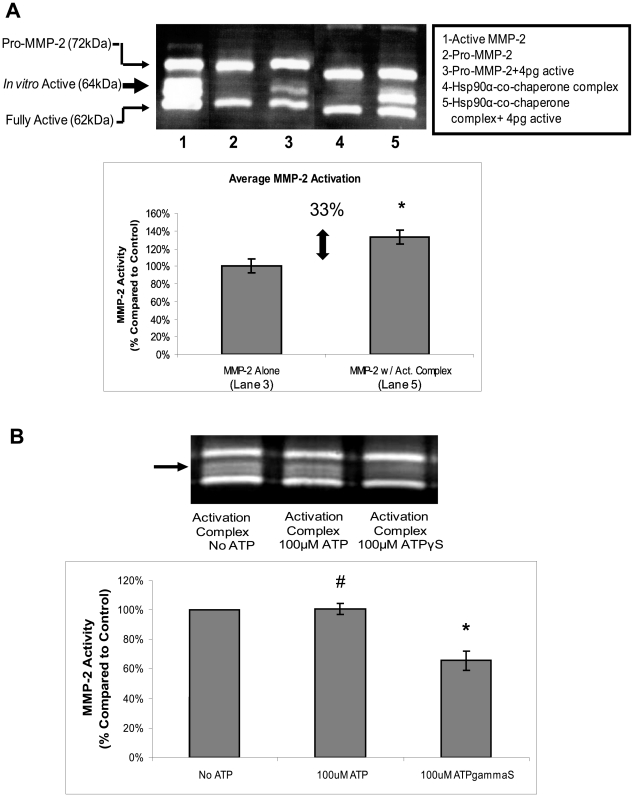
Hsp90α and co-chaperones Hsp70, Hop, Hsp40, and p23 are sufficient to activate MMP-2 *in vitro*. (a) 0.5 µg Pro-MMP-2 was incubated with and without 4 pg of activated MMP-2 and was incubated with 1 µg Hsp90α, 1 µg Hsp70, 0.25 µg Hop, 0.1 µg Hsp40, and 0.25 µg p23 recombinant proteins as indicated in the figure at 30°C for 30 minutes. 0.5 ug Pro-MMP-2 was activated in 1 mM APMA at 37°C for 2 hours (b) The same amounts of protein as above were incubated with 100 µM ATP, 100 µM ATPγS, or alone at 30°C for 30 minutes. The proteins were then added to a non-reducing sample buffer and then run on a gelatin containing SDS-PAGE. The gels were renatured for 40 minutes, digested for 18 hours and stained with Coomassie Brilliant Blue. Hsp90α Chaperone Complex = Hsp90α, MMP-2, Hsp70, Hop, Hsp40, and p23. Experiments were repeated three times. (a) P-value<0.05 (b) #P-value>0.3, *P-value<0.05.

Previous studies on Hsp90α and MMP-2 activation were preformed with conditioned media, which contains many proteins including active MMP-2. It was not known if Hsp90α alone could activate MMP-2 *in vitro*. In the following experiments, MMP-2 activity was assessed by measuring the relative amount of digestion seen in a gelatin zymogram. When MMP-2 is activated in an *in vitro* environment three bands are observed: the 72 kDa band is pro-MMP-2; the 64 kDa band is an intermediate, active form that is generated during *in vitro* activation; and the 62 kDa band represents the normal sized active MMP-2. Activation of MMP-2 in a completely *in vitro* environment, such as the above experiment, results in an active, 64 kDa, isoform, as can be seen in Fig. 4a [18], [28], [29]. We then investigated if pro-MMP-2 can be activated by Hsp90α, Hsp70, Hop, Hsp40, and p23 *in vitro*. When these proteins were added to pro-MMP-2, no activation was observed (Fig. 4). However, when we combined this mixture with a very small amount of activated MMP-2 (4 pg), cleavage and activation of pro-MMP-2 occurred. We observed a significant increase in the generation of the 64 kDa band (33% p<0.05) when Hsp90α and the co-chaperones were present, compared with MMP-2 alone (Fig. 4a, Figure S1a). To show that Hsp90α and the individual co-chaperones are important in this activation, we removed Hsp90α, Hsp70, or Hsp40 from the complex and in each instance saw a significant decrease in the amount of activated MMP-2 (Figure S1b). We also used a FITC-Casein assay to quantitatively measure the activation of MMP-2. We found the level of activation to be very similar (38%) to that detected in the zymogram (Figure S1c).

These findings demonstrate that the added co-chaperones can assist in MMP-2 activation *in vitro* and suggests a role for the complex in facilitating active MMP-2 cleavage of pro-MMP-2. The amount of activation observed is modest (33%), perhaps because there are additional components in the extracellular media that further increase the efficiency of the MMP-2 activation *in vivo*. For example, while we observed *in vitro* activation of MMP-2 in the absence of ATP, ATP could potentially increase the rate of MMP-2 activation as it does for the intracellular client proteins of Hsp90α. Therefore, we tested if ATP could enhance activation by combining the Hsp90α-co-chaperone complex proteins as described above, with 100 µM ATP, 100 µM ATPγS or no ATP and measuring the amount of MMP-2 activity. We found that neither ATP nor ATPγS increased MMP-2 activity and that ATPγS decreased MMP-2 activity (Fig. 4b). These results indicate that ATP does not enhance Hsp90α-mediated MMP-2 activation. This supports our hypothesis that extracellular Hsp90α functions with co-chaperones to assist in the activation of MMP-2 and can do so independently of ATP.

### Inhibiting Hsp70 reduces MMP-2 activation

**Figure 5:**
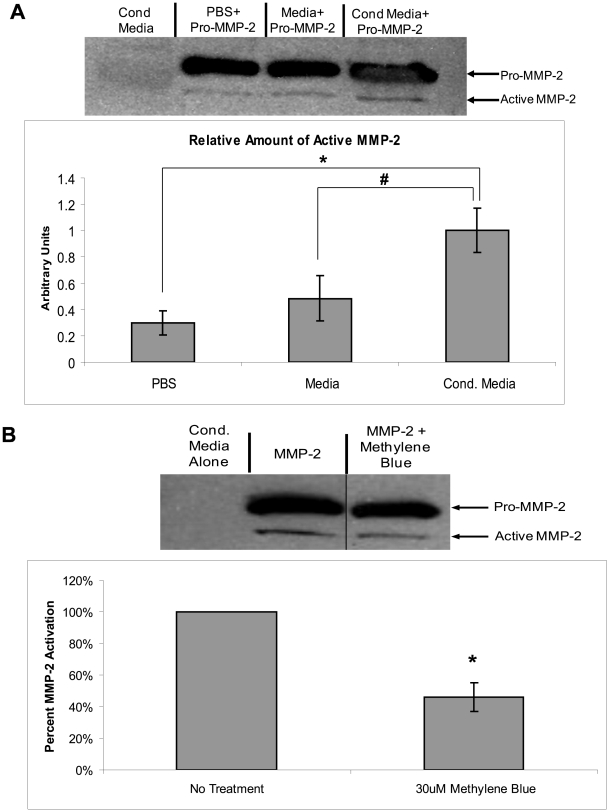
Conditioned media from cancer cells contains necessary components to activate MMP-2. (a) Conditioned media was collected from MDA-MB-231 cells, samples were incubated with and without 0.75 µg pro-MMP-2 for 5 minutes and the relative amount of active MMP-2 was analyzed via SDS-PAGE. (b) Conditioned media was collected and then treated with 30 µM Methylene Blue. 0.75 µg recombinant pro-MMP-2 was added to treated and untreated conditioned media. Samples were incubated at room temperature for 5 minutes and the relative amount of active MMP-2 was analyzed via SDS-PAGE. The difference in overall band intensity was taken into account in the densitometry calculations. Experiments were repeated three times. (* P-value<0.01, #P-value<0.05).

We next investigated whether the endogenous Hsp90α and co-chaperones present in the extracellular media of breast cancer cells can activate MMP-2. First, we demonstrated that the conditioned media collected from MDA-MB-231 cells contain the necessary components to activate MMP-2. In brief, recombinant pro-MMP-2 was added to PBS, untreated media, or MDA-MB-231 conditioned media. Then the amount of active MMP-2 was measured by SDS-PAGE and western blotted for MMP-2. Western blotting is a more accurate way of assessing the amount of active MMP-2 than zymography when comparing levels of active MMP-2 from samples which may contain TIMPs, which can interfere with MMP-2 activity [30]. When cellular components are present, as in the conditioned media experiments in Fig. 5a, MMP-2 is processed to the fully active 62 kDa band and the middle 64 kDa band is usually not seen [31]. As shown in Fig. 5a, the MMP-2 added to the conditioned media has 3.5-fold (P<0.01) greater activity than MMP-2 added to PBS and 2.5-fold (P<0.05) greater activity than MMP-2 added to untreated media. The amount of active MMP-2 is much greater than the amount of MMP-2 present in the conditioned media (conditioned media alone, lane 1), so the increase in MMP-2 activity is not the result of endogenous MMP-2. The amount of MMP-2 in the control lane is significantly less than that shown in figure one due to a lower exposure time for the film and the use of less conditioned media in the blot.

We then tested if Hsp70, a component of the co-chaperone complex, contributed to the observed MMP-2 activation by using an Hsp70 inhibitor, Methylene Blue [32]. When conditioned media was subject to Methylene Blue treatment, MMP-2 activation was reduced by 53% (p<0.01) (Fig. 5b). These findings show that Methylene Blue can reduce MMP-2 activation in the conditioned media, suggesting a role for Hsp70 in MMP-2 activation in breast cancer cells. It is possible that other proteases could influence MMP-2 activation, but the significant decrease in MMP-2 activation when Hsp70 was inhibited suggests that the Hsp90α-activation complex plays an important role in this activation.

### Extracellular Hsp90α and Hsp70 increase breast cancer cell migration

**Figure 6:**
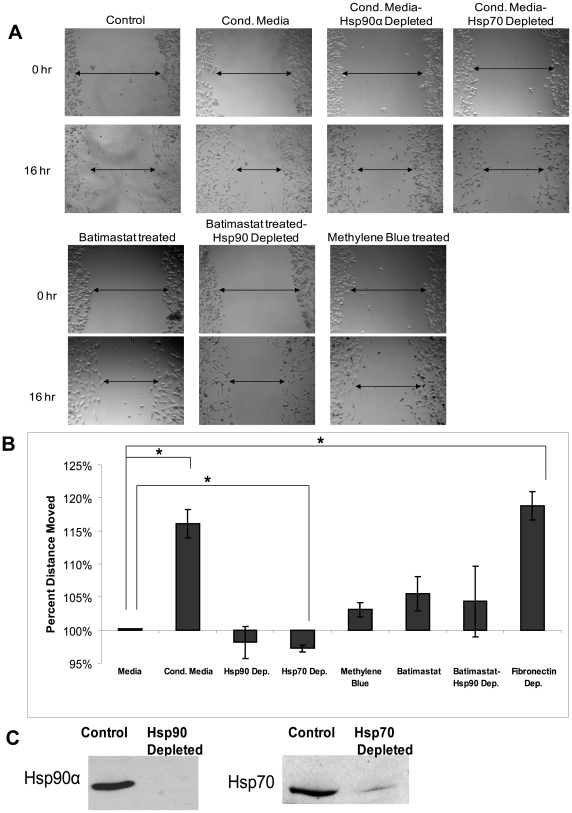
Hsp90α and its co-chaperone complex assist in wound healing. MDA-MB-231-s4175 cells were plated in an 8-well chamber slide and allowed to grow to confluency. A wound was created in the monolayer and either media, untreated conditioned media, Hsp90α depleted conditioned media, Hsp70 depleted conditioned media, 40 nM Batimastat (an MMP inhibitor) treated conditioned media, or 30 µM Methylene Blue (an Hsp70 inhibitor) treated conditioned media were added to the cells. The cells were incubated for 16 hours, and then wound closure was observed. (a) Representative pictures of wounded cells at 0 hours and 16 hours. (b) Graph of relative movement of cells. (c) SDS-PAGE showing Hsp90α and Hsp70 depletion. Experiment was repeated three times. (*P-value<0.01).

We next investigated the ability of the Hsp90α activation complex, specifically Hsp70, to increase breast cancer cell migration using a wound healing assay. MMPs are central in cancer cell invasion due to their ability to digest components of the extracellular matrix, allowing cancer cells to intravasate into the bloodstream [33]. MMPs have also been implicated in cancer cell migration. For instance, MMP-2 can cleave the adhesive contacts and cellular networks that cells use to adhere to their basement membrane, facilitating cell migration [14], [34], [35]. Therefore, we hypothesized that extracellular Hsp90α and Hsp70 increase cancer cell migration. To test this, we performed wound healing assays with MDA-231-s4175 cells, a derivative of the MDA-MB-231 cell line. MDA-MB-231 cells move slowly in *in vitro* wound healing assays and are thus not suited for this assay, whereas the s4175 derivative of the MDA-MB-231 cell line is highly motile in this assay. The MDA-MB-231 s4175 cell line was selected for its ability to metastasize to the lungs in a mouse model by Massagué et al. [36] Prior to the wound healing assays we verified that all of the co-chaperones identified in the original MDA-MB-231 line were also present in this sub-line (Figure S2). MMP-2 and all the co-chaperones examined are found at higher levels in the conditioned media of s4175 cells compared with media from the parent line, consistent with the high invasiveness of these cells. Wounds were created in monolayers of MDA-231-s4175 cells and incubated with either untreated media or conditioned media collected from other MDA-231-s4175 cells. Wound healing was measured after 16 hours. Cells that had been fed conditioned media migrated 16% (p<0.01) further than control cells that were fed with normal media (Fig. 6a and b). Therefore additional extracellular proteins present in conditioned media significantly increased the rate of cell migration. When we immunodepleted either Hsp90α or Hsp70 (Fig. 6c) the observed increase in migration from the conditioned media disappeared; the wound size did not significantly differ from the wound size of the control media-treated cells. Roles for Hsp90α and Hsp70 in conditioned media are further supported using inhibitors of these chaperones. When 17AAG or Methylene Blue [32] was added to the conditioned media there was a similar reduction in wound healing. To verify that the reduction in migration is due to the effect of Hsp90α on MMP-2 activation, and not Hsp90α affecting some other factor, we compared undepleted conditioned media treated with 40 nM Batimastat (a pan-MMP inhibitor) with Hsp90α-depleted conditioned media treated with 40 nM Batimastat to wounded cells. There was no significant difference in the reduction in migration between the two conditions, indicating that the effect of Hsp90α depletion and MMP inhibition is not additive (Fig. 6). A proliferation assay was performed to verify that the above conditions did not alter the cells' rate of proliferation (data not shown).

### Inhibiting Hsp70 reduces breast cancer cell invasion

**Figure 7:**
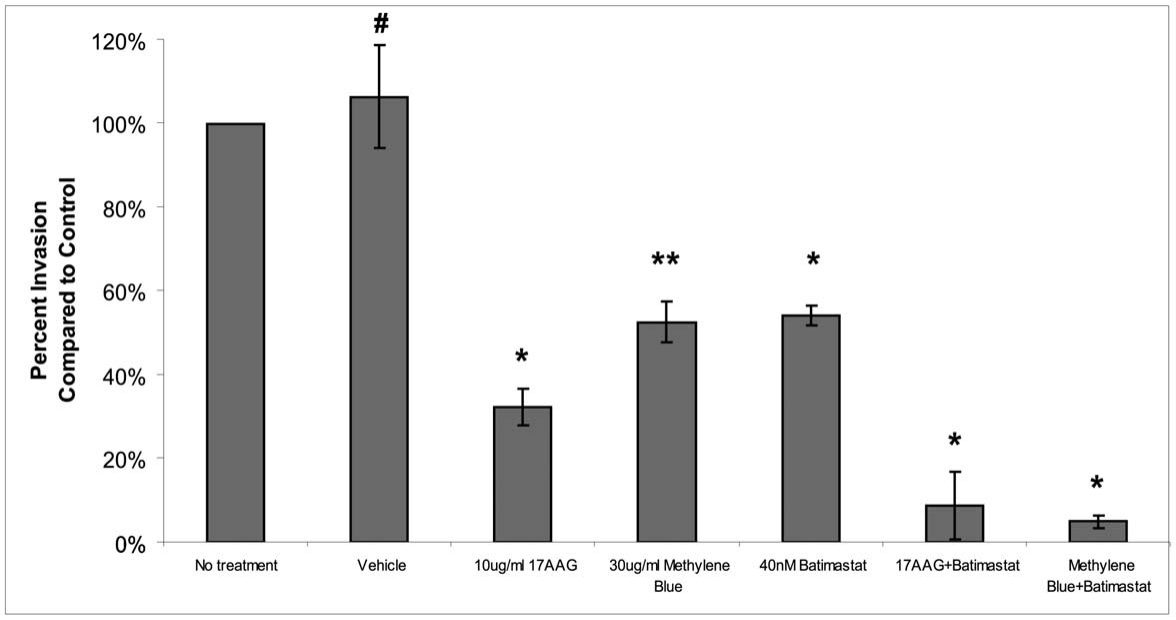
Inhibiting Hsp70 reduces breast cancer cell invasion *in vitro*. We performed invasion assays on MDA-MB-231 breast cancer cells to test the Hsp90α inhibiting compound 17AAG, the Hsp70 inhibiting compound Methylene Blue and the pan-MMP inhibitor Batimastat in a Chemotex invasion assay. We treated cells with 10 µg/ml 17AAG, 30 µg/ml water (vehicle), 30 µg/ml Methylene Blue or 40 nM Batimastat as indicated in the figure. Experiments were repeated three times. (*P<0.01,**P<0.05, #P>0.05).

Tumor cell invasion through the basement membrane and extracellular matrix that surrounds cancer cells is a key step in cancer cell metastasis and requires several cellular processes including migration and extracellular matrix remodeling [3], [37]. As Hsp70 increases Hsp90α-mediated MMP-2 activation and cell migration, we hypothesized that inhibition of Hsp70 would reduce cell invasion. Cells treated with 30 ug/ml of Methylene Blue saw a 50% decrease in invasion (p<0.05). Cells treated with vehicle showed no decrease in invasion (Fig. 7). Methylene Blue is cell-permeable, and can therefore also affect intracellular Hsp70. When cells in our invasion assay were treated with 40 nM Batimastat, we observed a similar decrease in invasion as seen with the Hsp70 or Hsp90α inhibitors. Combining Batimastat with the Hsp70 or Hsp90α inhibitors, we saw additive effects on invasion. These findings suggest that while Hsp70 and Hsp90α affect migration primarily via MMP-2 activation, they may have other roles in invasion. This is consistent with recent literature demonstrating that other proteins required for invasiveness are activated by Hsp70 and Hsp90α [7], [12]. The difference between migration and invasion is not surprising as invasion requires additional cellular processes than migration. Taken together, these findings indicate that Hsp90α and Hsp70 assist in MMP-2 activation, which increases breast cancer cell migration and contributes to breast cancer invasion.

## Discussion

Extracellular Hsp90α has been implicated in activating a number of proteins important for disease- relevant processes [38]. While the mechanism of Hsp90α activation of intracellular client proteins is well understood, this is not yet known for extracellular Hsp90α. Here we showed that extracellular Hsp90α binds to these co-chaperones both *in vitro* and in the conditioned media of MDA-MB-231 breast cancer cells. Moreover, the presence of these co-chaperones increases MMP-2 binding to Hsp90α and assists in MMP-2 activation *in vitro* and in conditioned media. Depletion or inhibition of Hsp70 significantly decreases MMP-2 activation, migration and invasion. Together, these findings implicate Hsp70 as part of a co-chaperone complex that acts with Hsp90α in MMP-2 activation and contributes to breast cancer migration and invasion.

Recently, Song et al. (2010) showed that extracellular Hsp90α can stabilize MMP-2, a process important for angiogenesis [39]. We did not observe the same MMP-2 degradation products as Song et al. indicating the identification of a different mechanism than the one described by their group (Figure S3). The different cell types and processes addressed in the two studies suggest that there may be multiple mechanisms for MMP-2 activation that may depend on what other proteins are present extracellularly. In this paper we show that extracellular Hsp90α, in conjunction with co-chaperones including Hsp70, assists in MMP-2 activation in an ATP-independent manner. It is well established that intracellular Hsp90α works together with co-chaperones to activate its client proteins, and we provide evidence suggesting that extracellular Hsp90α functions in a similar way. Intracellular Hsp90α has a minimum number of co-chaperones required for the activation of its client proteins *in vitro*: Hsp70, Hop, Hsp40, and p23 [5], [8]. Here, we demonstrated that these co-chaperones are present outside of MDA-MB-231 breast cancer cells.

Our findings suggest a role for Hsp70 in MMP-2 activation, as well as in the migration and invasion of breast cancer cells. While Methylene Blue is cell permeable and, can thus also inhibit intracellular Hsp70, antibody-based immunodepletion experiments implicate extracellular Hsp70 in these cancer-relevant processes. While extracellular Hsp70 has been identified outside immune cells, where it activates both the innate and adaptive immune system by interacting with antigen-presenting cells [40], its role in cancer cells and invasion is not yet clear [41]. Thus, our findings implicate a second chaperone, Hsp70, in MMP-2 activation and breast cancer cell migration and invasion. In addition, several of the other co-chaperones that we identified outside of MDA-MB-231 cells were shown to be in HT-1080 fibrosarcoma cell media [26]. Similar to our results, this study also did not find Hip in HT-1080 conditioned media; however they also did not find Hsp70. The absence of Hip likely does not impinge on the proposed mechanism because it is not required for the activation of most Hsp90α client proteins [8]. We tested for Hsp70 in HT-1080 conditioned media and did observe its presence (data not shown). The difference between these results is likely due to the use of better antibodies in the current study (Cell Cycle. 2010 Dec 1;9(23):4769).

Our studies have focused on MMP-2 in breast cancer cells, but extracellular Hsp90α may be important for other cancers as well [12]. Many proteases that are secreted in their inactive form are likely required for invasion and Hsp90α may also activate these proteases. Recently, we identified plasmin as a second client protein for extracellular Hsp90α in breast cancer and glioblastoma conditioned media. Extracellular Hsp90α activates plasmin in conjunction with tissue plasminogen activator and annexin II [12]. Hsp90α has been shown to be important in other cancers as well. Extracellular Hsp90α is upregulated in malignant melanomas [42], colorectal cancer [43] and fibrosarcomas [13], and is shown to increase heregulin-induced Her-2 activation and signaling [7]. Outside of the cell, Hsp90α has been shown to have a role in eliciting a host immune response against various antigens [44], [45], to activate plasminogen in smooth muscle cells during oxidative stress [46], and increase wound healing in human dermal fibroblasts [47]. Extracellular Hsp90α is also found in neurons and Schwann cells during development [48] and in TGF-alpha stimulated keratinocytes [49]. While the mechanism of extracellular Hsp90α in these additional roles is not known, we speculate that the role we identified for Hsp90α and Hsp70 in MMP-2 activation may have bearing on the role of Hsp90α in these other processes.

Interestingly, we did not find ATP to be necessary for MMP-2 activation, even though it is known to increase the reaction efficiency for intracellular Hsp90α function. This may be due to the fact that all of the conformational changes Hsp90α undergoes in its chaperoning processes can be accessed without the presence of ATP. Furthermore, recent work indicates that ATP is not necessary for Hsp90α to carry out its chaperoning function [50]. In addition we demonstrated that ATPγS decreased MMP-2 activity. This may occur because when ATP is bound by Hsp90α it must be hydrolyzed in order to evoke a conformation change to release its client protein. Binding a slowly hydrolysable form of ATP would reduce this transition and hence reduce the amount of MMP-2 activation. ATP has been found to be present outside of the cell in sub-micromolar concentrations as an extracellular ATP gradient [51], [52]; however, these low concentrations are unlikely to affect the function of extracellular Hsp90α. Our results are consistent with a recent finding that the extracellular Hsp90α stabilization of MMP-2 is ATP-independent [39]. In addition, Hsp70, which we demonstrated to be an essential co-chaperone in the Hsp90α-mediated activation of MMP-2, is also known to be an ATPase. But as noted above, we were able to obtain MMP-2 activation without the assistance of ATP. There is little literature on the mechanism of Hsp70 outside of the cell and whether it functions with the assistance of ATP. Hsp70 has a high affinity for ADP and therefore needs a high concentration of ATP to initiate the ATPase cycle which would be difficult to obtain outside of the cell. In addition, Hsp70 has been found to have a high level of conformational plasticity, especially in the nucleotide binding domain, possibly assisting in an ATP-independent extracellular function [53]. Our results, combined with other findings regarding Hsp70 function, suggest the existence of an ATP-free mechanism for extracellular Hsp70. We previously demonstrated that Hsp90α is exported via exosomes, but it is not known how the other co-chaperones are exported [12]. Hsp70 has been shown to be exported via exosomes in immune cells so it is possible that Hsp70 and the other co-chaperones are exported in a similar manner in breast cancer cells.

In this paper we identified an Hsp90α-co-chaperone complex that is necessary to activate MMP-2 *in vitro* and in cell culture. We identified the minimum components needed to obtain activation, but it is likely that there are other proteins that make the activation process more efficient. Since Hsp90α has been shown to activate other extracellular proteins [12], [47], it would be interesting to determine if this activation complex exists in normal cells as well as cancer cells and if it assists in the activation of other extracellular client proteins. Hsp90α and Hsp70 may be found to chaperone or activate other proteins using a similar mechanism to the activation for MMP-2. As such, these extracellular proteins may be targets for discovering drugs to prevent breast cancer invasion and metastasis.

## Supporting Information

Figure S1 (a and b) 0.5 µg Pro-MMP-2 and 4 pg of activated MMP-2 were incubated with 1 µg Hsp90α, 1 µg Hsp70, 0.25 µg Hop, 0.1 µg Hsp40, and 0.25 µg p23 recombinant proteins as indicated in the figure at 30°C for 0, 5, 15, and 30 minutes. The proteins were then added to a non-reducing sample buffer and run on a gelatin containing SDS-PAGE. The gels were renatured for 40 minutes, digested for 18 hours and stained with coomassie blue. Hsp90α Chaperone Complex = Hsp90α, MMP-2, Hsp70, Hop, Hsp40, and p23. (c) A FITC-Casein Assay was performed according to the manufactures directions except that the incubation buffer was substituted with 50 mM Tris–HCl, pH 7.5, 0.1 M NaCl, 10 mM CaCl_2_, and 0.1% Brij-35. Results were normalized to the control. *P-value<0.01.
(TIF)

Figure S2 Lysate and conditioned media were collected from MDA-MB-231(s4175) cells. 20 µg of protein was loaded into each lysate lane and 80 µg of protein was loaded into each conditioned media lane and immunoblotted for the Hsp90α, MMP-2, Hsp70, Hop, Hsp40, and p23. The molecular weight markers are indicated on the left.
(TIF)

Figure S3 PBS, Serum-free media, or conditioned media collected from MDA-MB-231 cells were incubated with and without 0.75 µg pro-MMP-2 for 5 minutes at room temperature and the relative amount of active MMP-2 was analyzed via SDS-PAGE.
(TIF)
