# Supplementary figures and images for: Extracellular Heat Shock Protein (Hsp)70 and Hsp90α Assist in Matrix Metalloproteinase-2 Activation and Breast Cancer Cell Migration and Invasion
Source: PLoS One. 2011 Apr 14;6(4):e18848. doi: 10.1371/journal.pone.0018848 (PMC3077417; doi:10.1371/journal.pone.0018848)

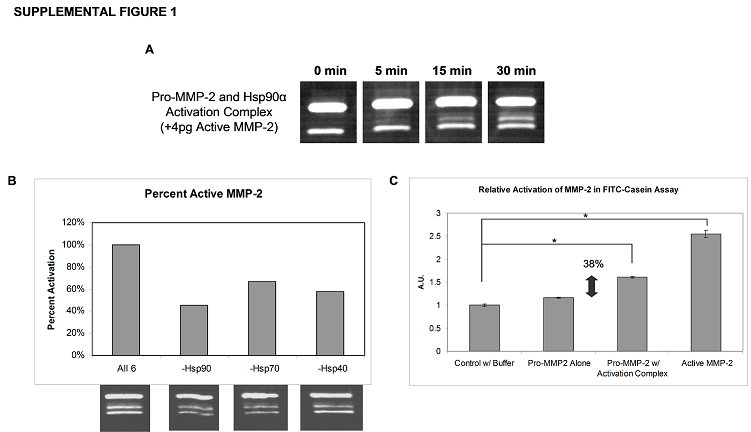

Supplement: Figure S1 — (a and b) 0.5 µg Pro-MMP-2 and 4 pg of activated MMP-2 were incubated with 1 µg Hsp90α, 1 µg Hsp70, 0.25 µg Hop, 0.1 µg Hsp40, and 0.25 µg p23 recombinant proteins as indicated in the figure at 30°C for 0, 5, 15, and 30 minutes. The proteins were then added to a non-reducing sample buffer and run on a gelatin containing SDS-PAGE. The gels were renatured for 40 minutes, digested for 18 hours and stained with coomassie blue. Hsp90α Chaperone Complex = Hsp90α, MMP-2, Hsp70, Hop, Hsp40, and p23. (c) A FITC-Casein Assay was performed according to the manufactures directions except that the incubation buffer was substituted with 50 mM Tris–HCl, pH 7.5, 0.1 M NaCl, 10 mM CaCl2, and 0.1% Brij-35. Results were normalized to the control. *P-value<0.01. (TIF) [file pone.0018848.s001.tif]

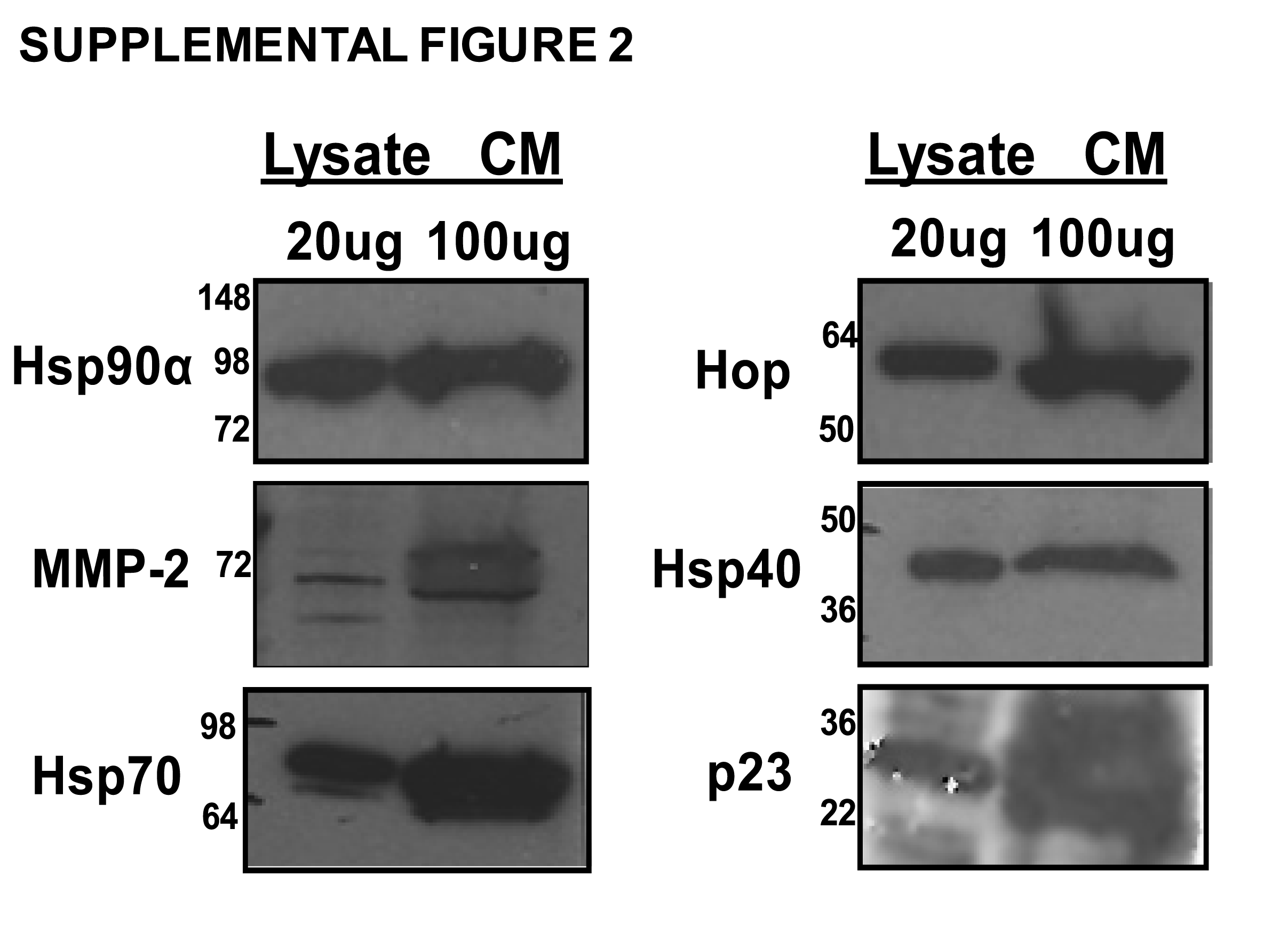

Supplement: Figure S2 — Lysate and conditioned media were collected from MDA-MB-231(s4175) cells. 20 µg of protein was loaded into each lysate lane and 80 µg of protein was loaded into each conditioned media lane and immunoblotted for the Hsp90α, MMP-2, Hsp70, Hop, Hsp40, and p23. The molecular weight markers are indicated on the left. (TIF) [file pone.0018848.s002.tif]

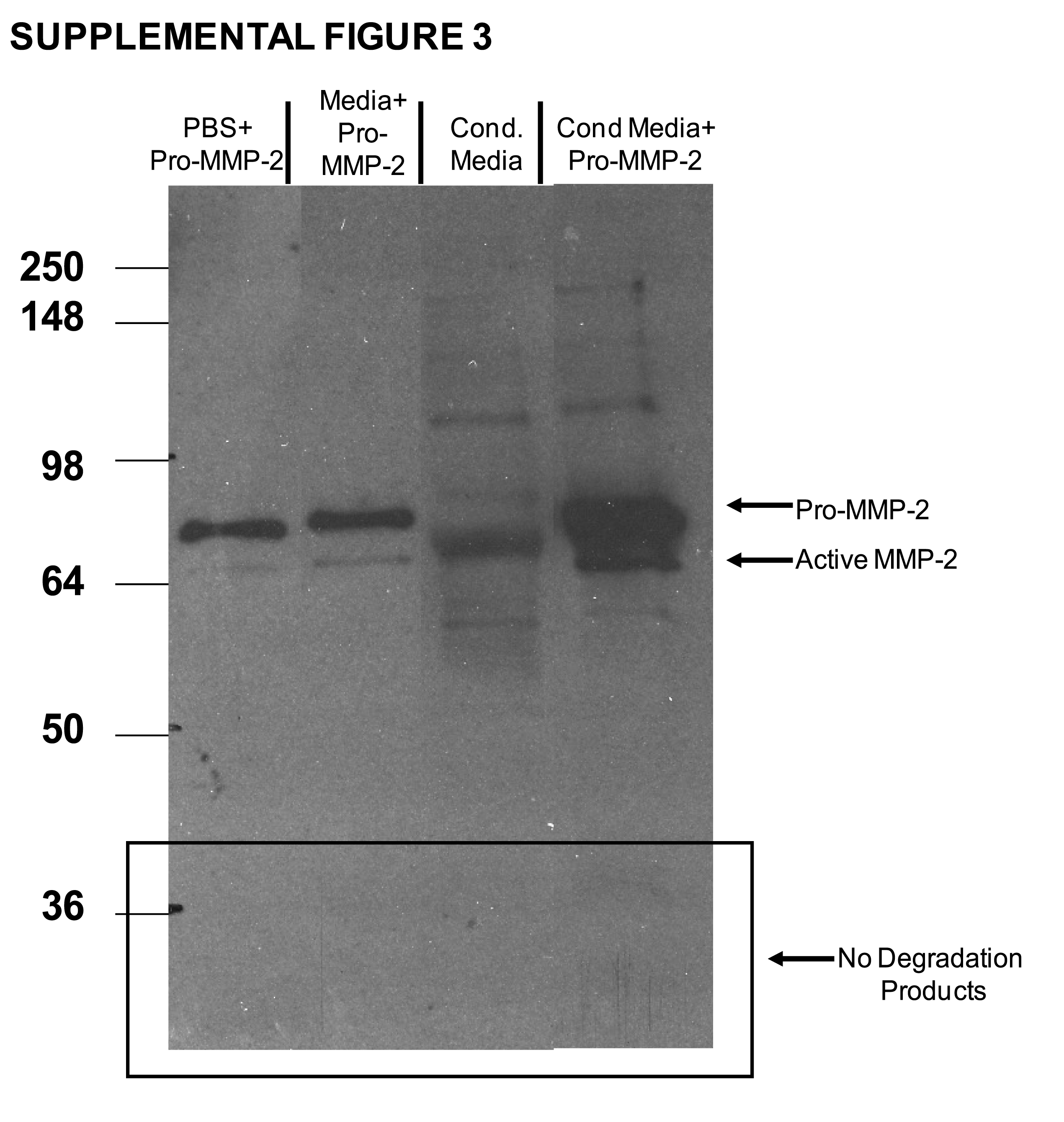

Supplement: Figure S3 — PBS, Serum-free media, or conditioned media collected from MDA-MB-231 cells were incubated with and without 0.75 µg pro-MMP-2 for 5 minutes at room temperature and the relative amount of active MMP-2 was analyzed via SDS-PAGE. (TIF) [file pone.0018848.s003.tif]
